# WHO guidelines for a healthy diet and mortality from cardiovascular disease in European and American elderly: the CHANCES project^1^,^2^

**DOI:** 10.3945/ajcn.114.095117

**Published:** 2015-09-09

**Authors:** Nicole Jankovic, Anouk Geelen, Martinette T Streppel, Lisette CPGM de Groot, Jessica C Kiefte-de Jong, Philippos Orfanos, Christina Bamia, Antonia Trichopoulou, Paolo Boffetta, Martin Bobak, Hynek Pikhart, Frank Kee, Mark G O’Doherty, Genevieve Buckland, Jayne Woodside, Oscar H Franco, M Arfan Ikram, Ellen A Struijk, Andrzej Pajak, Sofia Malyutina, Růžena Kubinova, Maria Wennberg, Yikyung Park, H Bas Bueno-de-Mesquita, Ellen Kampman, Edith J Feskens

**Affiliations:** 3 Division of Human Nutrition, Wageningen University, Wageningen, Netherlands;; 4 Centre of Clinical Epidemiology, Institute for Medical Informatics, Biometry and Epidemiology, University Hospital, University Duisburg-Essen, Essen, Germany;; 5 Department of Epidemiology, Erasmus MC, University Medical Centre, Rotterdam, Netherlands;; 6 Global Public Health, Leiden University College, the Hague, Netherlands;; 7 Department of Hygiene, Epidemiology and Medical Statistics, University of Athens, Medical School, Athens, Greece;; 8 Hellenic Health Foundation, Athens, Greece;; 9 The Tisch Cancer Institute, Mount Sinai School of Medicine, New York, NY;; 10 Department of Epidemiology and Public Health, University College London, London, United Kingdom;; 11 UKCRC Centre of Excellence for Public Health, School of Medicine, Dentistry and Biomedical Sciences, Queens University Belfast, Belfast, United Kingdom;; 12 Unit of Nutrition, Environment and Cancer, Cancer Epidemiology Research Programme, Catalan Institute of Oncology, Barcelona, Spain;; 13 Department of Epidemiology, Julius Centre, Utrecht, Netherlands;; 14 Department of Epidemiology and Population Studies, Jagiellonian University, Krakow, Poland;; 15 Institute of Internal and Preventive Medicine, Siberian Branch of the Russian Academy of Medical Sciences, Novosibirsk, Russia;; 16 Novosibirsk State Medical University, Novosibirsk, Russia;; 17 National Institute of Public Health, Prague, Czech Republic;; 18 Department of Public Health and Clinical Medicine, Nutritional Research, Umeå University, Umeå, Sweden;; 19 Division of Cancer Epidemiology and Genetics, National Cancer Institute, Bethesda, MD;; 20 Department for Determinants of Chronic Diseases, National Institute for Public Health and the Environment, Bilthoven, Netherlands;; 21 Department of Gastroenterology and Hepatology, University Medical Centre, Utrecht, Netherlands;; 22 Department of Epidemiology and Biostatistics, The School of Public Health, Imperial College London, London, United Kingdom; and; 23 Department of Social & Preventive Medicine, Faculty of Medicine, University of Malaya, Kuala Lumpur, Malaysia

**Keywords:** aging, CHANCES, cardiovascular disease, cohort, meta-analysis

## Abstract

**Background:** Cardiovascular disease (CVD) represents a leading cause of mortality worldwide, especially in the elderly. Lowering the number of CVD deaths requires preventive strategies targeted on the elderly.

**Objective:** The objective was to generate evidence on the association between WHO dietary recommendations and mortality from CVD, coronary artery disease (CAD), and stroke in the elderly aged ≥60 y.

**Design:** We analyzed data from 10 prospective cohort studies from Europe and the United States comprising a total sample of 281,874 men and women free from chronic diseases at baseline. Components of the Healthy Diet Indicator (HDI) included saturated fatty acids, polyunsaturated fatty acids, mono- and disaccharides, protein, cholesterol, dietary fiber, and fruit and vegetables. Cohort-specific HRs adjusted for sex, education, smoking, physical activity, and energy and alcohol intakes were pooled by using a random-effects model.

**Results:** During 3,322,768 person-years of follow-up, 12,492 people died of CVD. An increase of 10 HDI points (complete adherence to an additional WHO guideline) was, on average, not associated with CVD mortality (HR: 0.94; 95% CI: 0.86, 1.03), CAD mortality (HR: 0.99; 95% CI: 0.85, 1.14), or stroke mortality (HR: 0.95; 95% CI: 0.88, 1.03). However, after stratification of the data by geographic region, adherence to the HDI was associated with reduced CVD mortality in the southern European cohorts (HR: 0.87; 95% CI: 0.79, 0.96; *I*^2^ = 0%) and in the US cohort (HR: 0.85; 95% CI: 0.83, 0.87; *I*^2^ = not applicable).

**Conclusion:** Overall, greater adherence to the WHO dietary guidelines was not significantly associated with CVD mortality, but the results varied across regions. Clear inverse associations were observed in elderly populations in southern Europe and the United States.

## INTRODUCTION

The prevention of cardiovascular disease (CVD)^24^ later in life is of increasing public health interest, because the number of elderly people is growing constantly and the occurrence of CVD increases with advancing age (1). Evidence on potential risk factors for disease development and mortality is limited and inconclusive for older adults. Therefore, consortia such as the Consortium on Health and Ageing: Network of Cohorts in Europe and the United States (CHANCES) have been formed to examine risk factors in an exclusively elderly population (aged ≥60 y) to provide evidence for the prevention of premature death.

Diet is an important modifiable risk factor for CVD incidence (2), even in the elderly (1, 3). To maximize the reduction of CVD through diet, evidence-based, country-specific dietary guidelines were formulated and operationalized into healthy diet scores. Examples for such dietary pattern indexes are the Healthy Eating Index of the United States (4) and the Dutch Healthy Eating Index (5), which are useful for investigating country-specific associations between dietary quality and CVD. Thus far, evidence from multiple countries on the association between a healthy diet, defined as the adherence to dietary recommendations, and CVD mortality was not comparable enough to perform a meta-analysis (6). However, such data are important for drawing a convincing conclusion on the benefits of a healthy diet on CVD.

Deriving comparable data on dietary quality across cohorts requires a globally applicable dietary quality score (7, 8). The Healthy Diet Indicator [HDI (9)], based on WHO’s 2003 (10) nutrient intake goals to prevent chronic diseases worldwide, represents a globally applicable diet quality index that has been shown to be associated with all-cause mortality (9, 11, 12). All WHO recommendations were set after a proper review of most recent literature on diet and health (10). The indicator includes recommendations on the intake of dietary fatty acids (which affect plasma lipids and lipoproteins), total carbohydrates and free sugars (which mainly affect body fatness), cholesterol (as a marker for animal products), protein (which potentially influences blood lipid concentrations, blood pressure, and body weight), sodium (which affects blood pressure), fruit and vegetables (which have anti-inflammatory and antioxidant effects), and dietary fiber (which affect insulin sensitivity, blood pressure, lipids, and inflammation) (10, 13).

The aim of this meta-analysis was to add to the current knowledge regarding the potential benefits of adhering to a healthy diet (HDI) by preventing CVD mortality in the elderly. Furthermore, we evaluated whether this association would differ by age, sex, and geographic location. The current analysis complements a previous study that we conducted within CHANCES on the association between a healthy diet and longevity using all-cause mortality as the outcome (11). In the current study, we focused on the benefits of a healthy diet regarding cause-specific CVD mortality. Most importantly, the number of included cohorts allowed the additional analysis of coronary artery disease (CAD) and stroke mortality, which has seldom been analyzed in previous studies.

## METHODS

We conducted an individual participant–based meta-analysis within CHANCES (www.chancesfp7.eu). Its aim is to combine and integrate prospective cohort studies to produce, improve, and clarify the evidence on the distribution and risks factors for chronic diseases in the elderly and their socioeconomic implications. *Elderly* were defined by the CHANCES consortium as being aged ≥60 y. The cohorts of CHANCES were chosen because they undertook the efforts to harmonize all variables needed for this project according to predefined rules. The harmonization rules were discussed among the CHANCES partners until a consensus was reached.

We included participants aged ≥60 y (according to the definition of elderly by CHANCES) from the European Prospective Investigation into Cancer and Nutrition Elderly study (EPIC-Elderly) (14) [Spain (ES), the Netherlands (NL), Greece (GR), and the northern part of EPIC-Elderly Sweden (SE)]; the Health, Alcohol and Psychosocial factors in Eastern European countries (HAPIEE) (15) [Czech Republic (CZ), Russia (RU), and Poland (PL)]; the NIH-AARP Diet and Health Study in the United States, which included the following US regions: California, Louisiana, Florida, Atlanta, North Carolina, New Jersey, Pennsylvania, and Detroit (16); the Rotterdam Study (17) [Netherlands (NL)]; and the Survey in Europe on Nutrition and the Elderly; a Concerted Action (SENECA) (18) [multicenter European Study (European Union; EU)]. Before conducting the analysis, we excluded participants with incomplete follow-up information relevant to the analysis. We also excluded participants with missing information on age, chronic diseases (CVD, diabetes, and cancer) at baseline, missing or implausible information on BMI (in kg/m^2^; if BMI >60 or <10), and an unknown cause of death. A total number of 281,874 (74% of the original source population, see **Supplemental Table 1**) remained for further analysis. The Rotterdam Study and NIH-AARP showed dietary intake outliers, which we removed by Box-Cox transformation (i.e., participants beyond twice the IQR above the 75th or below the 25th percentile of sex-specific Box-Cox transformed energy intake were excluded).

The main characteristics of the cohorts were described previously (14–16, 18–22) and are summarized in Supplemental Table 1. In all of the cohorts, the procedures followed were in accordance with the ethical standards of the responsible institutional or regional committee on human experimentation, and all participants gave written informed consent.

### CVD mortality

CVD causes of death were defined by the following International Classification of Diseases (ICD) codes: ICD8 (390–458), ICD 9 (390–459), and ICD10 (100–199). CAD was defined by the following codes: ICD8 (410–414), ICD 9 (410–414), and ICD10 (120–125); and stroke by the following codes: ICD8 (430–438), ICD 9 (430–438), and ICD10 (160–169). Missing values for specific causes of death were <8% across cohorts. Participants with unknown cause of death were excluded from the analysis (*n* = 39,259; 10%). Start of follow-up was defined as age at baseline, and end of follow-up was defined as age of the participant at last linkage with the death registry (Supplemental Table 1).

### Dietary assessment

Different dietary-assessment methods were applied in each cohort. Most cohorts applied a validated food-frequency questionnaire (FFQ) (14–16, 18, 20–22). SENECA and EPIC-Elderly ES used a validated dietary-history method (23). HAPIEE applied the Whitehall II Study FFQ (15). The dietary-assessment methods applied in each of the cohorts were considered to be valid and reproducible. More information on the validity and reproducibility can be found elsewhere (23–32). The total number of either FFQ or dietary-history items, reference periods, and interview or self-reported assessments differed across cohorts. Foods were translated into nutrients by using country-specific food-composition tables. The cohort-specific definition for the food group “fruit and vegetables” is given in Supplemental Table 1.

### HDI

Huijbregts et al. (9) introduced the HDI for assessing the level of dietary quality within a population according to the WHO dietary guidelines, as published in 1990 (33). We substituted the WHO guidelines published in 1990 with the updated 2003 WHO guidelines on diet and nutrition to prevent chronic disease (10). In addition, the initial dichotomous scoring system (9) was replaced by a continuous scoring system, because this deals more efficiently with between-person variation and can better reveal diet-disease associations (6, 34). WHO components (as updated in 2003) and scoring standards are shown in **Table 1**. All cohorts had information on 9 nutrients and 1 food group out of the 14 WHO goals. Five of the 10 cohorts (3 cohorts of the HAPIEE study plus the NIH-AARP study and the Rotterdam Study) had information on all 10 codable dietary intake goals. To increase comparability across cohorts and with previous publications (9), we focused on the following 7 HDI components, which were available in all cohorts: percentage of energy intake from SFAs, PUFAs, mono- and disaccharides, and protein; cholesterol (mg/d), fruit and vegetables combined (g/d); and either total dietary fiber or nonstarch polysaccharides (g/d). The intake of n−3 PUFAs, n−6 PUFAs, *trans* fatty acids, and sodium were not included in the score. Furthermore, as suggested before (9), we excluded total fat and total carbohydrates from the HDI score calculation to avoid duplicating weights for these 2 components. We excluded MUFAs, because the WHO guideline does not account for the intake of MUFAs. Dietary fiber was used for the HDI calculation in all cohorts except HAPIEE, for which only nonstarch polysaccharide was available. Free sugars were not available in all cohorts and were replaced by mono- and disaccharides. According to the WHO guidelines, all macronutrients were expressed as a percentage of energy intake. For the calculation of nutrient densities, we excluded energy provided by ethanol, as performed earlier (9).

**TABLE 1:** Operationalization of the HDI based on WHO’s 2003 guidelines: CHANCES^1^

HDI component	Standard for minimum score of 0 points	Standard for continuous scoring of 0 to 10 points	Standard for maximum score of 10 points^2^
Moderation components			
SFAs, % of energy^3^	>15	10–15^4^	0–10
Mono- and disaccharides, % of energy^3^,^5^	>30	10–30^4^	0–10
Cholesterol, mg/d	>400	300–400^4^	0–300
Moderation range components			
PUFAs, % of energy^3^	>10	0–6^6^	6–10
Protein, % of energy^3^	>20	0–10^6^ or 15–20^4^	10–15
Adequacy components			
Total dietary fiber, g/d^7^	0	0–25^6^	>25
Fruit and vegetables, g/d	0	0–400^6^	>400

1 WHO guidelines not scored because of overlap with included components: total fat, MUFAs, and total carbohydrates. WHO guidelines that were not scored because of a lack of information: n−3 PUFAs, n−6 PUFAs, *trans* fatty acids, and sodium. CHANCES, Consortium on Health and Ageing: Network of Cohorts in Europe and the United States; HDI, Healthy Diet Indicator.

2 Standard in accordance with WHO guidelines.

3 Excluding energy from alcohol.

4 The upper cutoff value at which a participant would score 0 points was based on the 85th percentile of the population’s intake distribution. Calculation of points for dietary intake between the upper limit and the standard intake for maximum number of points: 10 − (intake − recommendation upper limit) × (10 ÷ standard upper limit − recommendation upper limit).

5 Free sugars were replaced by mono- and disaccharides.

6 Calculation of points for dietary intake between the lower limit and the standard intake for maximum number of points: (intake ÷ standard lower limit) × 10.

7 The joint WHO/FAO guidelines of 2003 do not indicate clear fiber cutoff values. Fulfillment of the fruit and vegetable recommendation and consumption of whole grains should sum up to 20 g nonstarch polysaccharides, which equals ∼25 g dietary fiber. Fiber was not available for Health, Alcohol and Psychosocial factors in Eastern European countries (HAPIEE). Therefore, we applied nonstarch polysaccharides instead for that cohort with a standard maximum score of 20.

The HDI includes 3 different categories of guidelines (“moderation,” “moderation range,” and “adequacy”) with accompanying scoring systems. The maximum score of 10 points was allocated if the intake was in accordance with the WHO guideline. For the moderation category (SFAs, mono- and disaccharides, and cholesterol), participants with a higher intake than recommended received proportionally fewer points, with a minimum of 0 points at the upper limit. The upper limit was defined as the 85th percentile of the combined cohort-specific population distribution (35). The “moderation range” components (PUFAs, 6–10% of energy; protein, 10–15% of energy) were scored with a maximum of 10 points if intake was within the recommended range. A score of zero corresponded to an intake of zero at the lower limit or the >85th percentile at the upper end. For PUFAs, 85% of our participants met the WHO guidelines, i.e., the upper limit was included in the recommended range. Therefore, all participants with a PUFA intake above the recommended range received 0 points. For the “adequacy” components (fiber, >25 g/d; fruit and vegetables, >400 g/d), participants received 10 points if they met the guidelines, whereas participants with lower intakes were allocated proportionately fewer points, with 0 g/d as the minimum.

After all individual scores were summed, a participant would receive the maximum HDI score of 70 points if all of the guidelines were met and the minimum HDI score of 0 if none of the guidelines was met (36).

### Covariates

Sex, education, alcohol consumption, smoking status, and energy intake were assessed by study-specific questionnaires and were available for all cohorts. Data on measured weight and height were available for EPIC-Elderly, the Rotterdam Study, and SENECA; self-reported data were provided by the NIH-AARP and HAPIEE studies. In the Rotterdam Study, no baseline measure for physical activity was available. For participants of the Rotterdam Study, we used physical activity assessed 7 y after baseline as a proxy measure for physical activity at baseline. Physical activity, for participants dying within the first 7 y after baseline, was coded as missing. Data on physical activity in EPIC-Elderly SE was not available for this study and was therefore not included as a covariate for any analysis performed in EPIC-Elderly SE. The following variables were available in some but not all cohorts and, therefore, were additionally included in the multivariate model but not considered for the pooled analysis: use of lipid-lowering drugs was available in EPIC-Elderly GR and the Rotterdam Study, history of hypertension (self-reported or documented) was known for EPIC-Elderly (ES, NL, GR, and SE), the Rotterdam Study, and SENECA. Information on multivitamin use was available for the Rotterdam Study only. Potential confounders were selected on the basis of prior knowledge regarding their association with dietary patterns and CVD risk.

### Statistical analysis

This meta-analysis of individual participant data followed a 2-step approach by analyzing each of the 10 cohorts individually, first by using the same analysis script and thereafter by conducting meta-analyses of the obtained effect estimates.

All analyses were performed by using the same analysis script. Cox proportional hazard models, with age applied as the underlying time variable, were used to assess the association between the HDI score (per 10-point increment, equivalent to the adherence of an additional WHO recommendation and in agreement with the cohort-specific IQRs) and subsequent CVD, CAD, and stroke mortality. SENECA was analyzed as one cohort because of the low number of cases per participating country. The cohort-specific HRs were summarized by random-effects meta-analysis to take differences in sample size and the possibility of statistical heterogeneity among the studies into account. Between-study heterogeneity was judged by *I*^2^ statistics. *I*^2^ statistics should be interpreted as the level of inconsistency across HR estimates instead of the real variation across the underlying true effects (37). To verify that our result was not solely driven by NIH-AARP, we conducted a random-effects meta-analysis and additionally stratified by region.

The final HR was adjusted for sex, education (primary or less, more than primary but less than college or university, or college or university), alcohol consumption [low (0 g/d), medium (men >0–40 g/d and women >0–20 g/d), and high (men >40 g/d and women >20 g/d)], smoking status (never, former, or current), energy intake (kcal/d), and vigorous physical activity (yes or no). Participants with missing data for the confounding variables were included by a separate category for these variables. BMI was initially not included in the main model because of its potential influence on the association as an intermediate factor. However, to assess whether BMI had any influence on the pooled results, additional adjustment was performed in a sensitivity analysis. We included “center” for EPIC-Elderly multicenter cohorts (ES and NL) and “region” for SENECA in all models to adjust for potential differences in baseline hazards across centers or regions.

In an additional analysis, we ran models for the Rotterdam Study, EPIC-Elderly GR, and NIH-AARP, for which we had additional data available on hypertension at baseline, use of statins, and multivitamins. Inclusion of those variables did not change the hazard estimates to any material extent. To examine the importance of excluded HDI components (n−3 and n−6 PUFAs as separate components, *trans* fatty acids, and sodium) to the association between WHO guidelines and CVD mortality, we additionally investigated the complete HDI score based on 10 WHO components in HAPIEE, NIH-AARP, and the Rotterdam Study.

Potential effect modification by age, sex, BMI, smoking, education, and alcohol consumption was investigated in each cohort by including an interaction term between these variables and the HDI score. Furthermore, we stratified the results on a healthy diet and CVD mortality for potential effect modifiers to address heterogeneity. To examine possible sources of heterogeneity, we compared the pooled HR estimates and *I*^2^ values for CVD mortality with the CVD mortality estimates of stratified analyses. Stratified analyses by potential effect modifiers were limited to CVD mortality, because the numbers of CAD and stroke cases were too small for cohort-specific subgroup analyses. For the analysis stratified by geographic region, we categorized SENECA cohorts into northern [Belgium, Denmark, France (Hagenau), Netherlands, and Switzerland (Burgdorf)] and southern [France (Romans), Greece, Italy, Portugal, Spain, and Switzerland (Yverdon and Bellinzona)] European countries. EPIC-elderly ES and GR were classified as southern Europe, and EPIC-Elderly NL and SE were classified as northern Europe.

In a sensitivity analysis, we studied the influence of possible dietary changes after disease occurrence on HRs. We excluded participants who died within the first 2 y of follow-up, as performed earlier (38). Finally, to investigate the importance of specific HDI components, we excluded one HDI component at a time and included them as a covariable instead (39).

Cohort-specific data were analyzed by using SAS version 9.2. For the random-effects meta-analysis, the metafor package in R (version 2.15.0) was used. A *P* value <0.05 was considered to be statistically significant.

## RESULTS

**Table 2** shows the baseline characteristics of the 281,874 included CHANCES participants. A total of 3,322,768 person-years were accumulated across studies. During that time, 12,492, 6004, and 2401 people died of CVD, CAD, and stroke, respectively. The proportion of deaths due to CVD, CAD, and stroke was highest in SENECA (all participants aged ≥70 y), followed by the Rotterdam Study (longest follow-up). At baseline, the mean age ranged from 60 y in EPIC-Elderly SE to 73 y in SENECA (Table 2). The mean BMI ranged from 26 in the 2 northern European EPIC-Elderly cohorts (NL and SE) and the Rotterdam Study to 29 in EPIC-Elderly ES and GR. The median HDI scores (maximum: 70 points) ranged from 42 (IQR: 37–47) in HAPIEE (RUS and PL) to 54 (IQR: 49–59) in EPIC-elderly GR.

**TABLE 2:** Baseline characteristics, HDI, and components of 281,874 CHANCES participants^1^

	EPIC-Elderly (14)				Rotterdam Study (17)	HAPIEE (15)			NIH-AARP (16)	SENECA (18)
Variable	Spain(*n* = 4382)	Netherlands(*n* = 5711)	Greece(*n* = 7400)	Sweden(*n* = 3087)	Netherlands(*n* = 2970)	Czech Republic(*n* = 2345)	Russia(*n* = 2389)	Poland(*n* = 2639)	United States(*n* = 249,568)	Europe(*n* = 1383)
Start of follow-up	1992–1996	1993–1997	1994–1999	1992–1996	1989–1993	2002–2005	2002–2005	2002–2005	1995–1996	1988
End of follow-up	2009	2009	2011	2009	2010	2011	2010	2009	2008	1998
Person-years	58,287	74,024	77,923	58,287	44,309	18,628	18,314	18,630	2,942,034	12,332
Deaths										
Cardiovascular disease, *n* (%)	123 (3)	208 (4)	567 (8)	124 (4)	521 (18)	66 (3)	182 (6)	33 (1)	10,498 (4)	170 (12)
Coronary artery disease, *n* (%)	54 (1)	61 (1)	173 (2)	66 (2)	79 (3)	28 (1)	107 (4)	14 (1)	5366 (2)	56 (4)
Stroke, *n* (%)	29 (1)	67 (1)	180 (2)	26 (1)	158 (5)	9 (0)	61 (2)	4 (0)	1811 (1)	56 (4)
Women, *n* (%)	2493 (57)	5451 (95)	4559 (62)	1681 (54)	1846 (62)	1295 (55)	1563 (55)	1361 (52)	108,536 (43)	734 (53)
Age at baseline, y	63 ± 2^2^	64 ± 3	67 ± 5	60 ± 1	69 ± 6	65 ± 3	65 ± 3	65 ± 3	65 ± 3	73 ± 2
BMI, kg/m^2^	29 ± 4	26 ± 4	29 ± 5	26 ± 4	26 ± 4	28 ± 4	28 ± 4	27 ± 4	27 ± 4	27 ± 4
Education, *n* (%)										
Primary or less	3741 (85)	1862 (33)	6726 (91)	1678 (54)	1050 (35)	327 (14)	492 (17)	422 (16)	1797 (1)	921 (66)
More than primary	314 (7)	3176 (56)	405 (6)	1018 (33)	1687 (57)	1664 (71)	1609 (57)	1492 (57)	65,170 (26)	357 (26)
College or university	277 (6)	640 (11)	239 (3)	365 (12)	215 (7)	344 (15)	738 (26)	722 (27)	175,263 (70)	110 (8)
Smoking status, *n* (%)										
Never	2949 (67)	2734 (48)	5131 (69)	1870 (61)	1073 (36)	1209 (52)	1894 (67)	1300 (49)	90,634 (36)	763 (55)
Former	690 (16)	1928 (34)	1220 (16)	630 (20)	1271 (43)	659 (28)	361 (13)	747 (28)	122,634 (49)	381 (28)
Current	738 (17)	1021 (18)	839 (11)	508 (16)	606 (20)	459 (20)	584 (22)	582 (22)	26,796 (11)	239 (17)
Alcohol consumption, *n* (%)										
No	1760 (40)	1178 (21)	2410 (33)	400 (13)	550 (19)	846 (36)	2122 (75)	1799 (68)	56,446 (23)	488 (35)
Medium	2012 (46)	3743 (66)	4598 (62)	2686 (87)	2114 (71)	1191 (51)	660 (23)	594 (23)	167,751 (67)	707 (51)
High	610 (14)	790 (14)	392 (5)	1 (0)	306 (10)	268 (11)	56 (2)	223 (8)	25,371 (10)	180 (13)
Vigorously physically active, *n* (%)	227 (5)	3201 (56)	1574 (21)	NA	692 (23)	1624 (69)	890 (31)	1841 (70)	120,064 (48)	492 (36)
Energy intake, kcal/d	1937 ± 602	1720 ± 423	1791 ± 547	1616 ± 587	1886 ± 444	1968 ± 679	2414 ± 739	2118 ± 683	1786 ± 742	2007 ± 624
Total HDI score (maximum 70 points)^3^	46 (40, 51)	45 (40, 49)	54 (49, 59)	46 (41, 51)	44 (39, 48)	47 (42, 53)	42 (37, 47)	42 (37, 47)	53 (47, 57)	47 (42, 53)

1 CHANCES, Consortium on Health and Ageing: Network of Cohorts in Europe and the United States; EPIC-Elderly, European Prospective Investigation into Cancer and Nutrition elderly study; HAPIEE, Health, Alcohol and Psychosocial factors in Eastern European countries; HDI, Healthy Diet Indicator; NA, not applicable; SENECA, Survey in Europe on Nutrition and the Elderly; a Concerted Action.

2 Mean ± SD (all such values).

3 Values are medians; IQR in parentheses.

**Tables 3** and **4** show the overall HDI scores and their components for the lowest and highest HDI quartile per cohort. For most single HDI items, the difference in intake between the lowest and highest quartiles were as expected. However, across cohorts, differences in associations with the HDI score were observed for PUFAs and mono- and disaccharides. A positive association between HDI and mean PUFA intake—in a comparison of the highest with the lowest HDI quartile—was observed in EPIC-Elderly (NL and SE), the Rotterdam Study, and HAPIEE (PL); an inverse association in EPIC-Elderly (ES and GR), HAPIEE (CZ), NIH-AARP, and SENECA; and no association in HAPIEE (RUS). In addition, we observed a positive association between HDI and mean mono- and disaccharide intake in EPIC-Elderly (NL), the Rotterdam Study, HAPIEE (CZ), and NIH-AARP; an inverse association was found in EPIC-Elderly (ES, GR, and SE) and SENECA, and no association was found in HAPIEE (RUS and PL).

**TABLE 3:** HDI scores and their components by the lowest and highest HDI quartile in CHANCES: EPIC-Elderly and the Rotterdam Study^1^

	EPIC-Elderly (14)								Rotterdam Study (17)	
	Spain		Netherlands		Greece		Sweden		Netherlands	
Variable	Q1	Q4	Q1	Q4	Q1	Q4	Q1	Q4	Q1	Q4
*n*	1095	1095	1427	1428	1849	1850	771	771	742	742
HDI score, points	35 ± 4^2^	55 ± 3	36 ± 4	52 ± 3	44 ± 4	62 ± 2	37 ± 3	54 ± 3	36 ± 3	52 ± 3
SFAs, % of energy	13.5 ± 3.5	9.2 ± 2.1	15.8 ± 2.4	12.6 ± 2.5	14.0 ± 2.2	10.2 ± 1.4	16.2 ± 3.0	12.0 ± 2.4	16.5 ± 2.9	13.4 ± 3.0
PUFAs, % of energy	6.3 ± 3.5	5.6 ± 1.8	5.7 ± 2.1	7.0 ± 1.5	8.8 ± 4.5	5.4 ± 1.6	3.9 ± 0.8	4.6 ± 1.1	6.7 ± 3.8	7.6 ± 1.8
Protein, % of energy	20.9 ± 3.3	18.6 ± 2.9	19.9 ± 2.7	16.2 ± 2.3	16.9 ± 1.7	14.4 ± 1.3	15.8 ± 2.5	14.5 ± 1.6	18.2 ± 3.1	16.2 ± 2.6
Mono- and disaccharides, % of energy	18.7 ± 6.7	18.4 ± 6.0	24.1 ± 5.7	27.3 ± 5.9	17.4 ± 4.4	16.7 ± 3.7	21.7 ± 5.5	21.1 ± 5.3	22.3 ± 5.9	23.5 ± 5.9
Cholesterol, mg/d	427.9 ± 151.4	249.5 ± 90.7	235.5 ± 99.4	178.8 ± 60.2	208.3 ± 105.9	140.0 ± 59.0	177.2 ± 108.2	156.9 ± 59.6	260.2 ± 90.2	207.7 ± 58.8
Fiber, g/d	21.0 ± 8.4	26.7 ± 7.9	18.1 ± 5.2	26.0 ± 5.2	17.7 ± 6.1	23.4 ± 6.4	13.6 ± 6.0	25.1 ± 6.1	14.1 ± 3.8	19.3 ± 4.0
Fruit and vegetables, g/d	471.1 ± 297.0	630.0 ± 282.7	281.0 ± 130.2	457.7 ± 173.5	651.5 ± 267.4	837.3 ± 290.2	141.6 ± 101.4	388.8 ± 190.1	390.2 ± 162.7	500.4 ± 149.4

1 CHANCES, Consortium on Health and Ageing: Network of Cohorts in Europe and the United States; EPIC-Elderly, European Prospective Investigation into Cancer and Nutrition elderly study; HDI, Healthy Diet Indicator; Q, quartile.

2 Mean ±SD (all such values).

**TABLE 4:** HDI scores and their components by the lowest and highest HDI quartile in CHANCES: HAPIEE, NIH-AARP, and SENECA^1^

	HAPIEE (15)						NIH-AARP (16)		SENECA (18)	
	Czech Republic		Russia		Poland		United States		Europe	
Variable	Q1	Q4	Q1	Q4	Q1	Q4	Q1	Q4	Q1	Q4
*n*	585	586	709	710	659	659	62,392	62,392	345	345
HDI score, points	37 ± 4^2^	57 ± 3	33 ± 4	53 ± 4	33 ± 3	62.2 ± 2	42 ± 5	60 ± 2	33 ± 4	54 ± 3
SFAs, % of energy	15.3 ± 3.5	11.5 ± 2.2	15.7 ± 2.2	12.1 ± 2.4	16.1 ± 2.3	10.2 ± 1.4	12.1 ± 3.3	8.5 ± 1.9	16.9 ± 3.8	10.8 ± 3.7
PUFAs, % of energy	6.8 ± 1.9	6.7 ± 1.5	8.7 ± 2.6	8.9 ± 2.4	4.7 ± 1.1	5.4 ± 1.6	7.9 ± 2.9	7.3 ± 1.4	6.7 ± 4.2	5.5 ± 2.8
Protein, % of energy	19.2 ± 2.9	15.7 ± 2.2	18.7 ± 2.5	15.9 ± 2.5	19.4 ± 2.1	14.4 ± 1.3	17.0 ± 3.5	14.7 ± 1.9	16.4 ± 3.6	14.3 ± 1.9
Mono- and disaccharides, % of energy	19.0 ± 6.2	21.9 ± 7.8	17.3 ± 4.8	17.5 ± 4.9	20.2 ± 5.6	16.7 ± 3.7	23.5 ± 8.4	24.6 ± 6.7	19.0 ± 7.2	18.3 ± 8.2
Cholesterol, mg/d	379.4 ± 154.2	223.2 ± 69.5	496.6 ± 178.5	290.1 ± 124.8	446.7 ± 173.1	140.0 ± 59.0	252.9 ± 158.2	165.8 ± 70.5	380.5 ± 132.8	229.0 ± 76.1
Fiber, g/d	16.3 ± 9.9	24.4 ± 14.3	15.8 ± 6.0	18.3 ± 6.3	17.5 ± 7.6	23.4 ± 6.4	14.7 ± 8.4	24.6 ± 8.3	17.0 ± 7.4	24.9 ± 11.5
Fruit and vegetables, g/d	503.5 ± 475.6	857.6 ± 756.4	385.4 ± 278.0	465.8 ± 275.7	453.5 ± 290.4	837.3 ± 290.2	479.7 ± 334.8	821.9 ± 389.9	483.6 ± 244.9	617.8 ± 273.1

1 CHANCES, Consortium on Health and Ageing: Network of Cohorts in Europe and the United States; HAPIEE, Health, Alcohol and Psychosocial factors in Eastern European countries; HDI, Healthy Diet Indicator; Q, quartile; SENECA, Survey in Europe on Nutrition and the Elderly; a Concerted Action.

2 Mean ± SD (all such values).

**FIGURE 1:**
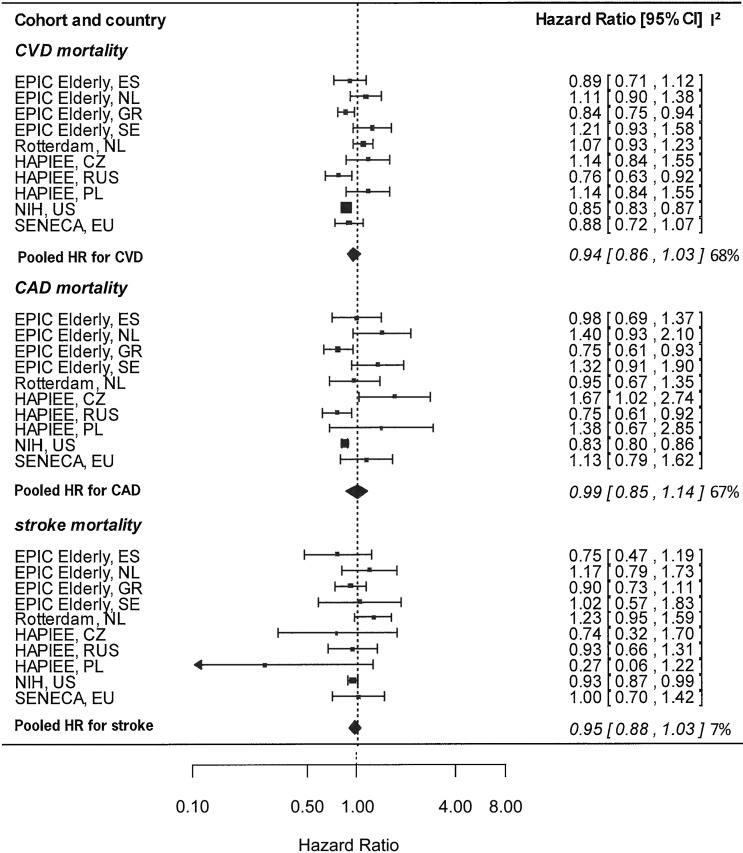
Cohort-specific and pooled HRs of CVD, CAD, and stroke mortality in relation to a 10-point increase in the Healthy Diet Indicator, adjusted for sex, education, smoking status, energy intake, alcohol consumption, and physical activity in CHANCES, 1988–2011. The bars represent 95% CIs. *I*^2^ values are expressed as a percentage of total variability due to heterogeneity. All data were obtained from the CHANCES consortium (www.chancesfp7.eu). Participants were from SENECA (18), the Rotterdam Study (17), EPIC-Elderly (14), NIH-AARP (16), and HAPIEE (15). CAD, coronary artery disease; CHANCES, Consortium on Health and Ageing: Network of Cohorts in Europe and the United States; CVD, cardiovascular disease; CZ, Czech Republic; EPIC-Elderly, European Prospective Investigation into Cancer and Nutrition elderly study; ES, Spain; EU, European Union; HAPIEE, Health, Alcohol and Psychosocial factors in Eastern European countries; GR, Greece; NL, Netherlands; PL, Poland; RUS, Russia; SE, Sweden; SENECA, Survey in Europe on Nutrition and the Elderly; a Concerted Action.

**Figure 1** shows the cohort-specific and pooled HRs for CVD, CAD, and stroke mortality per 10-point increase in the HDI (representing the adherence to an additional WHO guideline), after adjustment for sex, education, smoking status, energy intake, alcohol consumption, and physical activity. For CVD mortality, HRs per 10-point increases ranged from 0.84 for EPIC-Elderly GR to 1.21 for EPIC-Elderly SE. In the pooled analysis, on average, a nonsignificant reduction of 6% (HR: 0.94; 95% CI: 0.86, 1.03) in CVD mortality was observed, per 10-point increases in HDI. Heterogeneity was high (*I*^2^ = 68%). Additional adjustment for BMI did not influence the pooled HR estimate for CAD (HR: 0.94; 95% CI: 0.86, 1.03), CVD (HR: 0.95; 95% CI: 0.83, 1.09), and stroke (HR: 0.94; 95% CI: 0.89, 1.00).

For CAD mortality, HRs ranged from 0.75 for EPIC-Elderly GR and HAPIEE (RUS) to 1.40 for EPIC-Elderly NL; no association was observed across cohorts (HR: 0.99; 95% CI: 0.85, 1.14; *I*^2^ = 67%). HR estimates for stroke mortality ranged from 0.74 for HAPIEE (CZ) to 1.23 for the Rotterdam Study. Overall average risk reduction for stroke mortality amounted to 5% (HR: 0.95; 95% CI: 0.88, 1.03; *I*^2^ = 7%).

The significance levels for interaction derived in each cohort separately did not suggest effect modification. However, stratified analysis showed reduced levels of heterogeneity after stratification in most cases. For CVD deaths, the pooled HRs were similar for men and women (*I*^2^ = 56% for men and 63% for women) (**Table 5**). Participants aged ≥70 y showed a slightly stronger association (HR: 0.91) compared with the overall estimate (HR: 0.94). A significant inverse association (HR: 0.89; 95% CI: 0.83, 0.96; *I*^2^ = 24%) between HDI and CVD mortality was observed for participants with a BMI ≥27 but not for participants with a BMI <27. Significant inverse associations with low heterogeneity were also observed in former smokers, medium-level-educated subjects, and no or high alcohol users.

**TABLE 5:** HRs and 95% CIs stratified by potential effect modifiers and cohort-specific characteristics for the association of a 10-point increment in the Healthy Diet Indicator and mortality due to CVD, CAD, and stroke: CHANCES^1^

Outcome variable and strata	CVD deaths/participants, *n*	HR and 95% CI^2^	*I*^2^, %
Stratified analysis by potential effect modifiers of CVD only			
Sex			
Men	7938/152,804	0.93 (0.84, 1.04)	56
Women	4554/127,976	0.93 (0.82, 1.05)	63
Age group			
60–70 y	10,914/265,707	0.94 (0.84, 1.04)	72
>70 y	1576/1628	0.91 (0.83, 1.00)	19
BMI			
<27 kg/m^2^	6730/166,661	0.98 (0.87, 1.11)	66
≥27 kg/m^2^	5762/115,655	0.89 (0.83, 0.96)	24
Smoking			
Never	3528/109,543	0.95 (0.84, 1.08)	64
Former	5711/130,518	0.84 (0.81, 0.87)	0
Current	2709/32,371	0.93 (0.84, 1.04)	32
Education			
Primary or less	1339/19,002	0.91 (0.79, 1.05)	68
More than primary	3726/76,891	0.88 (0.84, 0.92)	0
College or university	7035/178,911	1.07 (0.78, 1.48)	55
Geographic region^3^			
CVD			
US	10,498/249,568	0.85 (0.83, 0.87)	NA
EU	1994/32,306	0.96 (0.87, 1.06)	55
CEE	281/7373	0.96 (0.70, 1.31)	67
Southern Europe	790/12,640	0.87 (0.79, 0.96)	0
Northern Europe	923/12,293	1.02 (0.85, 1.24)	63
CAD			
US	5366/249,568	0.83 (0.80, 0.86)	NA
EU	638/32,306	1.00 (0.85, 1.18)	52
CEE	149/7373	1.15 (0.64, 2.06)	79
Southern Europe	262/12,640	0.88 (0.72, 1.08)	44
Northern Europe	227/12,293	1.16 (0.94, 1.42)	0
Stroke			
US	6811/249,568	0.93 (0.87, 0.99)	NA
EU	590/32,306	0.99 (0.87, 1.12)	5
CEE	74/7373	0.80 (0.51, 1.24)	22
Southern Europe	248/12,640	0.90 (0.76, 1.08)	0
Northern Europe	268/12,293	1.14 (0.95, 1.35)	0
Additional analysis excluding participants who died within 2 y of follow-up			
CVD	11,482/266,860	0.95 (0.86, 1.04)	69
CAD	5501/272,841	0.99 (0.85, 1.14)	62
Stroke	2247/276,095	0.95 (0.89, 1.00)	0

1 CAD, coronary artery disease; CHANCES, Consortium on Health and Ageing: Network of Cohorts in Europe and the United States; CEE, central and eastern Europe; CVD, cardiovascular disease; EPIC-Elderly, European Prospective Investigation into Cancer and Nutrition elderly study; EU, European Union; HAPIEE, Health, Alcohol and Psychosocial factors in Eastern European countries; SENECA, Survey in Europe on Nutrition and the Elderly; a Concerted Action.

2 All models were adjusted for potential confounding variables: sex, education (primary or less, more than primary but less than college or university, or college or university), alcohol consumption [low (0 g/d), medium (men >0–40 g/d and women >0–20 g/d), or high (men >40 g/d and women >20 g/d)], smoking status (never, former, or current), energy intake (kcal/d), and vigorous physical activity (yes or no).

3 US = NIH-AARP (16); EU = HAPIEE (all; 15), EPIC-Elderly (all; 14), SENECA (18), and the Rotterdam Study (17); CEE = HAPIEE (all); southern Europe = EPIC-Elderly (Greece, Spain) and SENECA (south); northern Europe = EPIC-Elderly (Netherlands, Sweden), the Rotterdam Study, and SENECA (north).

The inclusion of additional WHO components in the HDI score, in the 5 cohorts with available data, showed estimates similar to the results derived in the main analysis. We observed a narrower CI but a greater level of heterogeneity (HR: 0.93; 95% CI: 0.80, 1.07; *I*^2^ = 81%) in contrast with the overall result derived in these 5 cohorts (HR: 0.94; 95% CI: 0.81, 1.09; *I*^2^ = 75%).

Stratification by geographic region showed a significant inverse association between the HDI and CVD mortality in the US (HR: 0.85; 95% CI: 0.83, 0.87) and southern European (HR: 0.87; 95% CI: 0.79, 0.96; *I*^2^ = 0%) cohorts but not in the central eastern European (HR: 0.96; 95% CI: 0.70, 1.31; *I*^2^ = 67%) and northern European (HR: 1.02; 95% CI: 0.85, 1.24; *I*^2^ = 63%) cohorts (Table 5). HDI showed a strong inverse association with CAD and stroke mortality in the United States and slightly stronger, albeit nonsignificant inverse, associations in the southern European cohorts compared with the overall pooled results for CAD and stroke. The northern European and central eastern cohorts showed no significant associations between HDI and any of the mortality outcomes. Exclusion of the first 2 y of follow-up showed similar results compared with the main analysis.

Finally, further sensitivity analyses were carried out to investigate the importance of the single HDI components by excluding them one at a time from the HDI and including them as a covariable instead (**Supplemental Table 2**). The analysis showed robust pooled HR estimates for CVD and stroke mortality, ranging from 0.93 for CVD and 0.94 for stroke (excluding SFAs, PUFAs, or mono- and disaccharides) mortality to 0.96 for CVD (excluding fruit and vegetables) and 0.97 for stroke mortality (excluding PUFAs and fruit and vegetables). HR estimates for CAD were less robust and mostly influenced by PUFAs (HR: 0.92) and cholesterol (HR: 0.91)

## DISCUSSION

Our study included 10 cohorts from Europe and the United States and comprised a total sample of 281,874 elderly participants, free of disease at baseline, with 12,492 CVD, 6004 CAD, and 2401 stroke deaths. The overall results for the association between the HDI guidelines and CVD, CAD, and stroke mortality showed, on average, no significant associations. HRs were similar in men and women, but varied across the BMI, smoking, alcohol use, and education categories. Geographic region appeared to be of main importance. Based on our data, the inverse association of HDI and CVD mortality appears convincing for the southern European countries and the United States, whereas the absence of an association in northern and central Eastern Europe was unexpected.

Previously, Huijbregts et al. (9) examined adherence to the 1990 WHO recommendations in men aged 50–70 y in relation to 20-y mortality. Participants from Finland, Italy, and the Netherlands—whose diet was in accordance with the WHO guidelines—had a significant 18% lower risk of dying from CVD compared with the group with the lowest adherence. In line with our findings for northern Europe, more recent studies showed no significant association between the HDI and CVD mortality in elderly men from Sweden (40) and the United Kingdom (41). The HDI includes subscores on SFAs, PUFAs, mono- and disaccharides, protein, cholesterol, fiber, and fruit and vegetables. As such, it was inversely associated with all-cause mortality in our CHANCES cohorts (HR: 0.90; 95% CI: 0.87, 0.93), with no evidence for regional variation regarding the direction of association (11). In our study, differences in food patterns across cohorts may have caused opposed associations between PUFAs and the HDI, which might partly explain the heterogeneous results in HR estimates between CVD mortality and all-cause mortality (11). For example, southern European diets (ES and GR) are characterized by a high consumption of plant foods, such as oils, whereas northern European diets (NL and SE) include a higher consumption of margarine, dairy products, sugar, potato, and processed meat (42). The composition of PUFAs within a dietary pattern may be more important in the context of CVD mortality than for all-cause mortality regarding the reduction in risk of premature death. However, the explanation for the observed difference in results for the association of the HDI and CVD with all-cause mortality and the reported regional differences of the current analysis remain speculative.

The northern European cohorts showed no association between WHO guidelines and CVD. One important food in the northern diet is margarine, which in the past was a potential source of *trans* fatty acids (43, 44) and was shown to increase the risk of CVD (43–45). The underlying food pattern of central eastern European countries might also have caused absent associations between the HDI and CVD mortality in the current study (20). One exception was HAPIEE RUS, which showed a significant inverse association for CVD mortality. NIH-AARP showed an HDI profile similar to that observed in southern European countries. Consequently, adherence to the HDI score was inversely associated with CVD mortality in NIH-AARP.

This points to a specific issue of the HDI, i.e., that it is mostly based on nutrient recommendations rather than on the consumption of foods. This makes sense, because it was intended to provide an overall recommendation globally, which could be locally adapted into food-based dietary guidelines for communication purposes. A side effect could be that the HDI is not an optimal indicator of a healthy diet in a specific population, as our data seem to suggest. On the other hand, our results show that—in the US and southern European cohorts—a 10-unit increment in the HDI (corresponding to the IQR) is associated with a reduction in CVD mortality of 13% to 15%. This indicates that the guidelines translated in dietary patterns frequently used in highly educated US seniors and elderly from southern Europe are likely to be beneficial from a cardiovascular point of view.

Our findings are based on a meta-analysis of individual participant data. Combining cohort studies in a meta-analysis typically results in a high level of heterogeneity (46). *I*^2^ values decreased considerably after stratification. Regional differences partly explained the level of heterogeneity, at least for the southern European countries. The level of heterogeneity within northern and central eastern Europe remained high and may have been related to differences in the study design and dietary-assessment methods used. Moreover, differences in the quality of health care systems (better medical support and screening systems) in some cohorts may have influenced the HR estimates on mortality, which may have resulted in a greater level of heterogeneity.

Divergent associations across smoking, alcohol use, and education categories showed small levels of heterogeneity due to large CIs overlapping all point estimates. The differences found in BMI were mainly driven by SENECA and EPIC-Elderly SE, which both presented a positive association in the low-BMI group and an inverse association in the high-BMI group, which could also be driven by chance findings.

Measurement error of dietary exposure was a major concern in the current study, as it is in all large cohort studies of diet and chronic disease. The methods used were validated, but—especially for the FFQs, which aim to rank subjects according to intake rather than to estimate absolute intake levels—a calibration step may be needed when different cohorts are combined (47). However, we took this problem into account by using a random-effects meta-analysis, in which subjects with low and high HDIs were compared within each cohort. Another limitation of our dietary information related to the lack of dietary intake data during follow-up. The assessments were done only once, at baseline, which assumed stable dietary patterns over time. Generally, a single dietary measurement at baseline is susceptible to misclassification of long-term dietary intake because of reporting bias and changes occurring in the diet (48, 49). Dietary intake may be quite stable in the elderly (50, 51); however, because repeated measures over time probably improve estimates of association (52, 53), our result is an underestimation rather than an overestimation of the reported HR estimate.

Note that one limitation of studying an elderly population may be the presence of selection bias and missing follow-up data by “unhealthy” participants. This may have caused an underestimation of the overall pooled results (54, 55). Participants with prevalent CVD at baseline were excluded to reduce reverse causation. Our sensitivity analysis, which excluded those participants who died within the first 2 y of follow-up, showed results similar to those of the main analysis, which also made reverse causation unlikely. Although we excluded prevalent cases of CVD, we assumed that the result—a healthy diet according to WHO guidelines is inversely associated with CVD mortality—could be extrapolated to the patient group, because dietary guidelines for CVD prevention and treatment are generally similar.

Major advantages of this meta-analysis were the use of harmonized variables and identical analysis scripts across cohorts, the use of large data sets, the diversity of the population, and access to the original data. Furthermore, the score construction was based on the average 85th percentile across cohorts. This allowed a sufficient level of variation in the HDI score across cohorts and not only for a specific subcohort on which a cutoff level could have been assigned. A drawback of most diet scores, including the HDI score, may be related to missing weighing factors per recommendation. However, our sensitivity analysis, which excluded one HDI component at a time, showed comparable HR estimates for CVD with different levels of precision, which indicated that each recommendation is equally important. Nevertheless, the inclusion of weighing factors may improve the validity of the HDI. Even though the HDI was shown to decrease the risk of premature death (9, 11, 12), more research on the HDI’s content and construct validity and reliability is a logical step for further analysis (56).

In conclusion, the results of this study show that a healthy diet, based on the WHO guidelines, is significantly associated with decreased CVD mortality in US and southern European elderly. However, nonsignificant associations found for the northern European countries are possibly attributable to a less healthy underlying food pattern in comparison with the US and southern European cohorts. Future studies using the HDI or other nutrient-based scores should additionally focus on the underlying food pattern of the studied population.

## Footnotes

24 Abbreviations used: CAD, coronary artery disease; CHANCES, Consortium on Health and Ageing: Network of Cohorts in Europe and the United States; CVD, cardiovascular disease; CZ, Czech Republic; EPIC-Elderly, European Prospective Investigation into Cancer and Nutrition elderly study; ES, Spain; EU, European Union; FFQ, food-frequency questionnaire; GR, Greece; HAPIEE, Health, Alcohol and Psychosocial factors in Eastern European countries; HDI, Healthy Diet Indicator; ICD, International Classification of Diseases; NL, Netherlands; PL, Poland; RU, Russia; SE, Sweden; SENECA, Survey in Europe on Nutrition and the Elderly; a Concerted Action.
